# Fuzzy Upper Bounds in Groupoids

**DOI:** 10.1155/2014/697012

**Published:** 2014-05-28

**Authors:** Sun Shin Ahn, Young Hee Kim, J. Neggers

**Affiliations:** ^1^Department of Mathematics Education, Dongguk University, Seoul 100-715, Republic of Korea; ^2^Department of Mathematics, Chungbuk National University, Cheongju 361-763, Republic of Korea; ^3^Department of Mathematics, University of Alabama, Tuscaloosa, AL 35487-0350, USA

## Abstract

The notion of a fuzzy upper bound over a groupoid is introduced and some properties of it are investigated. We also define the notions of an either-or subset of a groupoid and a strong either-or subset of a groupoid and study some of their related properties. In particular, we consider fuzzy upper bounds in Bin(*X*), where Bin(*X*) is the collection of all groupoids. Finally, we define a fuzzy-*d*-subset of a groupoid and investigate some of its properties.

## 1. Introduction

Iséki and Tanaka introduced two classes of abstract algebras: BCK-algebras and BCI-algebras [1, 2].

Neggers and Kim [3] introduced the notion of *d*-algebras which is a useful generalization of BCK-algebras and then investigated several relations between *d*-algebras and BCK-algebras as well as several other relations between *d*-algebras and oriented digraphs. Han et al. [4] defined a variety of special *d*-algebras, such as strong *d*-algebras, (weakly) selective *d*-algebras, and others. The main assertion is that the squared algebra (*X*; □, 0) of a *d*-algebra is a *d*-algebra if and only if the root (*X*; ∗, 0) of the squared algebra (*X*; □, 0) is a strong *d*-algebra. Recently, Kim et al. [5] explored properties of the set of *d*-units of a *d*-algebra. It was noted that many *d*-algebras are weakly associative, and the existence of nonweakly associative *d*/BCK-algebras was demonstrated. Moreover, we discussed the notions of a *d*-integral domain and a left-injectivity in *d*/BCK-algebras.

The notion of the semigroup (Bin(*X*), □) was introduced by Kim and Neggers [6]. Fayoumi [7] introduced the notion of the center *Z*Bin(*X*) in the semigroup Bin(*X*) of all binary systems on a set *X* and showed that if (*X*, •) ∈ *Z*Bin(*X*), then *x* ≠ *y* implies {*x*, *y*} = {*x*•*y*, *y*•*x*}. Moreover, she showed that a groupoid (*X*, •) ∈ *Z*Bin(*X*) if and only if it is a locally zero groupoid. Han et al. [8] introduced the notion of hypergroupoids (*H*Bin(*X*), □) and showed that (*H*Bin(*X*), □) is a supersemigroup of the semigroup (Bin(*X*), □) via the identification *x*↔{*x*}. They proved that (*H*Bin*(*X*), ⊖, [*∅*]) is a BCK-algebra.

## 2. Preliminaries

A *d-algebra * [3] is a nonempty set *X* with a constant 0 and a binary operation “∗” satisfying the following axioms:

(D1) *x*∗*x* = 0,
(D2) 0∗*x* = 0,
(D3) *x*∗*y* = 0 and *y*∗*x* = 0 imply *x* = *y* for all *x*, *y* ∈ *X*.

A BCK-algebra is a *d*-algebra *X* satisfying the following additional axioms:

(D4)  ((*x*∗*y*)∗(*x*∗*z*))∗(*z*∗*y*) = 0,
(D5)  (*x*∗(*x*∗*y*))∗*y* = 0 for all *x*, *y*, *z* ∈ *X*.

**Example (see [4])**

Consider the real numbers **R**, and suppose that (**R**; ∗, **e**) has the multiplication

(1) $$\begin{array}{c} x*y=\left(x-y\right)\left(x-e\right)+e. \end{array}$$

Then *x*∗*x* = *e*; *e*∗*x* = *e*; *x*∗*y* = *y*∗*x* = *e* yield (*x* − *y*)(*x* − *e*) = 0, (*y* − *x*)(*y* − *e*) = 0 and *x* = *y*, or *x* = *e* = *y*; that is, *x* = *y*; that is, (**R**; ∗, *e*) is a *d*-algebra.

Given a nonempty set *X*, we let Bin(*X*) the collection of all groupoids (*X*, ∗), where ∗ : *X* × *X* → *X* is a map and where ∗(*x*, *y*) is written in the usual product form. Given elements (*X*, ∗) and (*X*, •) of Bin(*X*), define a product “□” on these groupoids as follows:

(2) $$\begin{array}{c} \left(X,*\right)□\left(X,•\right)=\left(X,□\right), \end{array}$$

where

(3) $$\begin{array}{c} x\;□\;y=\left(x*y\right)•\left(y*x\right) \end{array}$$

for any *x*, *y* ∈ *X*. Using the notion, Kim and Neggers [6] showed the following theorem.

**Theorem (see [6])**

(Bin(*X*), □) is a semigroup; that is, the operation “□” as defined in general is associative. Furthermore, the left-zero semigroup is an identity for this operation.

## 3. Four Different Types of Fuzzy Subsets

In what follows, let *X* denote a groupoid unless otherwise specified.

**Definition**

Let (*X*, ∗, 0) be a groupoid. A map *μ* of *X* is called a* fuzzy upper bound* (or shortly, a fub) over (*X*, ∗, 0) if *μ*(0) ≥ *μ*(*x*) for all *x* ∈ *X*. A map *μ* of *X* is called a* fuzzy lower bound* (or shortly, a flb) over (*X*, ∗, 0) if *μ*(0) ≤ *μ*(*x*) for all *x* ∈ *X*.

**Example**

Let (*X*, ∗) be any groupoid and let 0 ∈ *X* such that *μ*(0) is the greatest element of *μ*(*X*): = {*μ*(*x*) | *x* ∈ *X*} where *μ* : *X* → [0,1]. Then *μ*(0) ≥ *μ*(*x*) for all *x* ∈ *X*; that is, *μ* is a fub over (*X*, ∗, 0).

Given a fub *μ* : *X* → [0,1] over (*X*, ∗, 0), we consider the following conditions: for any *x*, *y* ∈ *X*,

 (*A*)  *μ*(*x*∗*y*) = *μ*(*y*∗*x*) = *μ*(0) implies *μ*(*x*) = *μ*(*y*),
 (*B*)  *μ*(*x*∗*y*) = *μ*(*y*∗*x*) = *μ*(0) implies *x* = *y*,
 (*C*)  *μ*(*x*∗*y*) = *μ*(*y*∗*x*) implies *μ*(*x*) = *μ*(*y*),
 (*D*)  *μ*(*x*∗*y*) = *μ*(*y*∗*x*) implies *x* = *y*.

We have a diagram of implications as follows:

(4) (B)⟹(A)⇑⇑(D)⟹(C).

Thus we will look for counter examples to (*A*)⇒(*B*), (*B*)⇒(*D*), (*A*)⇒(*C*), and (*C*)⇒(*D*).

**Example**

(a) Let (*X*, ∗, 0) be a groupoid with |*X*| ≥ 2 and let *μ* : *X* → [0,1] be a constant map; that is, *μ*(*x*) = *α* for some *α* ∈ [0,1] for all *x* ∈ *X*. Then (*A*) holds as does (*C*). On the other hand, *x* ≠ *y* implies (*B*) fails as does (*D*). Thus we have countered examples to (*A*)⇒(*B*) and (*C*)⇒(*D*).

(b) Let *X* : = {0,1, 2,3} be a *d*-algebra with the following table:

(5) ∗012300000110212220333330.

Define a map *μ* : *X* → [0,1] by

(6) $$\begin{array}{c} \mu \left(x\right):=\{\begin{array}{cc} 1, & \text{if}\;\;x=0,\\ 0, & \text{if}\;\;x\neq 0. \end{array} \end{array}$$

It is easy to check that *μ* is a fub over (*X*, ∗, 0) having the condition (*B*). But *μ* does not have the condition (*D*), since *μ*(1∗2) = *μ*(2) = *μ*(2∗1) and 1 ≠ 2. Hence (*B*)⇒(*D*) is false.

(c) Let *X* = 2^*A*^, where *A* = {1,2, 3,4}. Define a binary operation “∗” on *X* by *x*∗*y* : = {*t* | *t* ∈ *x*, *t* ∉ *y*},  ∀*x*, *y* ∈ *X*. Thus, if *x*⊆*y*, then *x*∗*y* = *∅*. Let *μ*(*x*): = 1/(1 + |*x*|)  , where |*x*| is the cardinality of *x*. Assume *μ*(*x*∗*y*) = *μ*(*y*∗*x*) = *μ*(*∅*). Since *μ*(*∅*) = 1, we have |*x*∗*y*| = 0 = |*y*∗*x*| and so *x*∗*y* = *∅* = *y*∗*x*. Therefore *x*⊆*y*, *y*⊆*x*. Hence *x* = *y*. On the other hand, if *x* = {1,2, 3}, *y* = {3,4, 5}, then *x*∗*y* = {1,2}, *y*∗*x* = {4,5}, and *μ*(*x*∗*y*) = 1/3 = *μ*(*y*∗*x*), but *x* ≠ *y*. This shows that (*B*) is true, but (*D*) is false.

(d) Let (*X*, ∗, 0) be a left-zero-semigroup; that is, for any *x*, *y* ∈ *X*, *x*∗*y* = *x*, and let *μ* : *X* → [0,1] be a fub over (*X*, ∗, 0). Assume that *μ*(*x*∗*y*) = *μ*(*y*∗*x*) for any *x*, *y* ∈ *X*. Since (*X*, ∗, 0) is a left-zero-semigroup, we have *μ*(*x*) = *μ*(*x*∗*y*) = *μ*(*y*∗*x*) = *μ*(*y*). Hence condition (*C*) holds for any fub *μ* over (*X*, ∗, 0).

(e) Let (*X*, ∗, 0) = (*R*, −, 0), where *R* is the set of all real numbers and “−” is the usual subtraction on *R*. Also, if *μ*(*x*) = *e* ^−*x*^2^^, then *μ*(0) = 1 ≥ *μ*(*x*) for all *x* ∈ *R*; that is, *μ* is a fub over (*R*, −, 0). Note that *μ*(*x* − *y*) = *μ*(*y* − *x*) = *μ*(0) implies *x* − *y* = *y* − *x* = 0 and *x* = *y*, so that *μ*(*x*) = *μ*(*y*) as well; that is, the conditions (*B*) and (*A*) hold. Now if we let *y* : = −1 and *x* : = 2 + *y*, then *μ*(*x* − *y*) = *μ*(2 + *y* − *y*) = *μ*(2) = *e* ^−4^ and *μ*(*y* − *x*) = *μ*(*y* − 2 − *y*) = *μ*(−2) = *e* ^−4^, so that *μ*(*x* − *y*) = *μ*(*y* − *x*). Since *e* ^−4(*y*+1)^ < 1 for any *y* ≠ −1, we have *μ*(*x*) = *μ*(2 + *y*) = *e* ^−(2+*y*)^2^^ = *e* ^−*y*^2^^ *e* ^−4(*y*+1)^ < *e* ^−*y*^2^^ = *μ*(*y*). Thus (*C*) fails to hold.

As a refinement of the condition (*A*), we give two conditions (*A*)* and (*A*)_α_*, 0 ≤ *α* ≤ 1 as follows:

 (*A*)*  *μ*(*x*∗*y*) = *μ*(*y*∗*x*) = *μ*(0) implies *μ*(*x*) = *μ*(*y*) = *μ*(0),
 (*A*)_α_*  *μ*(*x*∗*y*) = *μ*(*y*∗*x*) = *μ*(0) implies *μ*(*x*) = *μ*(*y*) ≥ *α*.

**Example**

(a) If *μ* : *X* → [0,1] is the constant map, that is, *μ*(*x*) = *α* for all *x* ∈ *X*, then for any groupoid (*X*, ∗, 0)  *μ* satisfies the condition (*A*)*. Since (*A*)*⇒(*A*), *μ* satisfies the condition (*A*).

(b) Let (*X*, ∗, 0): = (*R*, −, 0), where *R* is the set of all real numbers and “−” is the usual subtraction on *R*, and let *μ*(*x*): = *e* ^−*x*^2^^. Then *μ*(0) = 1 ≥ *μ*(*x*) > 0 for any *x* ∈ *R*, and *μ*(*x*) = 1 if and only if *x* = 0. Assume that *μ*(*x* − *y*) = *μ*(*y* − *x*) = *μ*(0). Then *e* ^−(*x*−*y*)^2^^ = *e* ^−(*y*−*x*)^2^^ = 1 and so *x* − *y* = 0. Hence *x* = *y*. Therefore *μ*(*x*) = *μ*(*y*); that is, (*A*) holds. Let *x* = *y* ≠ 0 imply *μ*(*y*) = *μ*(*x*) = *e* ^−*x*^2^^ < 1 = *μ*(0). Thus (*A*)* does not hold. Since lim⁡_*x*→*∞*_ *μ*(*x*) = 0, given *α* > 0, there is an *x* such that *μ*(*x*) < *α*; that is, (*A*)_α_* does not hold for *α* > 0.

(c) For the groupoid (*R*, −, 0), let *μ*_*β*_(*x*): = (*e* ^−*x*^2^^ + *β*)/(1 + *β*), where *β* ≥ 0. Then *μ*_*β*_(0) = 1 ≥ *μ*_*β*_(*x*) for all *x* ∈ *R* and lim⁡_*x*→*∞*_ *μ*_*β*_(*x*) = *β*/(1 + *β*) ≤ *μ*_*β*_(*x*) for all *x* ∈ *X*. If we take *α* : = *β*/(1 + *β*), then *μ*_*β*_(*x*) ≥ *α* for all *x* ∈ *X*. If we assume *μ*_*β*_(*x* − *y*) = *μ*_*β*_(*y* − *x*) = *μ*_*β*_(0), then (*x* − *y*)^2^ = (*y* − *x*)^2^ = 0 and hence *μ*_*β*_(*x*) = *μ*_*β*_(*y*); that is, the condition (*A*)_α_* holds. If *α*_1_ < *α*_2_, then (*A*)_*α*_2__*⇒(*A*)_*α*_1__* holds clearly. If we set *β*_1_ : = *α*_1_/(1 − *α*_1_) and *β*_2_ : = *α*_2_/(1 − *α*_2_), then *β*_1_ < *β*_2_ and lim⁡_*x*→*∞*_ *μ*_*β*_1__(*x*) = lim⁡_*x*→*∞*_((*e* ^−*x*^2^^ + *β*_1_)/(1 + *β*_1_)) = (*β*_1_)/(1 + *β*_1_) = *α* < *α*_2_. Take an *x* ∈ *R* so that *α*_1_ < *μ*_*β*_1__(*x*) < *α*_2_. Then *e* ^−*x*^2^^ + *β*_1_ < *α*_2_(1 + *β*_1_). It follows that *e* ^−*x*^2^^ < (*β*_2_/(1 + *β*_2_))(1 + *β*_1_) − *β*_1_ < (*β*_2_(1 + *β*_1_) − *β*_1_(1 + *β*_2_))/(1 + *β*_2_) = (*β*_2_ − *β*_1_)(1 + *β*_1_)/(1 + *β*_2_); that is, *x* ^2^ > ln⁡[(1 + *β*_2_)/(*β*_2_ − *β*_1_)(1 + *β*_1_)]. Hence, if $\left|x\right|>\sqrt{\ln[(1+\beta_{2})/\left(\beta_{2}-\beta_{1}\right)\left(1+\beta_{1}\right)]}$, then (*A*)_*α*_1__*⇒(*A*)_*α*_2__* does not hold.

**Theorem 7**

Suppose that *μ*_1_ and *μ*_2_ are *fub*s over a groupoid (*X*, ∗, 0) and that they both satisfy the condition (*A*). If *μ* : = *λμ*_1_ + (1 − *λ*)*μ*_2_, where 0 ≤ *λ* ≤ 1, then *μ* is also a *fub* over (*X*, ∗, 0) having the condition (*A*).

**Proof**

Let 0 < *λ* < 1. For any *x* ∈ *X*, we have *μ*(0) = *λμ*_1_(0)+(1 − *λ*)*μ*_2_(0) ≥ *λμ*_1_(*x*)+(1 − *λ*)*μ*_2_(*x*) = *μ*(*x*). Hence *μ* is a fub over (*X*, ∗, 0).

Assume that *μ*(*x*∗*y*) = *μ*(*y*∗*x*) = *μ*(0), but *μ*_1_(*x*∗*y*) < *μ*_1_(0) or *μ*_2_(*x*∗*y*) < *μ*_2_(0). If *μ*_1_(*x*∗*y*) < *μ*_1_(0), then *λμ*_1_(*x*∗*y*) < *λμ*_1_(0) and thus (1 − *λ*)*μ*_2_(*x*∗*y*) = *μ*(*x*∗*y*) − *λμ*_1_(*x*∗*y*) > *μ*(*x*∗*y*) − *λμ*_1_(0) =  *μ*(0) − *λμ*_1_(0) = (1 − *λ*)*μ*_2_(0). This shows that *μ*_2_(*x*∗*y*) > *μ*_2_(0), which is a contradiction. Similarly, *μ*_2_(*x*∗*y*) > *μ*_2_(0) implies *μ*_1_(*x*∗*y*) > *μ*_1_(0), which is also a contradiction. Hence we obtain *μ*_1_(*x*∗*y*) = *μ*_1_(0), *μ*_2_(*x*∗*y*) = *μ*_2_(0). Similarly, we obtain *μ*_1_(*y*∗*x*) = *μ*_1_(0), *μ*_2_(*y*∗*x*) = *μ*_2_(0). Since *μ*_*i*_ has the condition (*A*), we have *μ*_*i*_(*x*) = *μ*_*i*_(*y*)  (*i* = 1,2). Hence *μ*(*x*) = *λμ*_1_(*x*)+(1 − *λ*)*μ*_2_(*x*) = *λμ*_1_(*y*)+(1 − *λ*)*μ*_2_(*y*) = *μ*(*y*). Thus *μ* has the condition (*A*).

**Theorem 8**

Suppose that *μ*_1_ and *μ*_2_ are *fub*s over a groupoid (*X*, ∗, 0) and that *μ*_2_ has the condition (*A*)* with *μ*_1_(0) < *μ*_2_(0). If *μ* : = *μ*_1_∨*μ*_2_, where (*μ*_1_∨*μ*_2_)(*x*): = max⁡{*μ*_1_(*x*), *μ*_2_(*x*)}, then *μ* is a *fub* over (*X*, ∗, 0) having the condition (*A*)*.

**Proof**

If *μ* = *μ*_1_∨*μ*_2_, then *μ*(0) = *μ*_1_(0)∨*μ*_2_(0), *μ*(*x*) = *μ*_1_(*x*)∨*μ*_2_(*x*). If *μ*_1_(*x*) ≥ *μ*_2_(*x*), then *μ*(*x*) = *μ*_1_(*x*) and *μ*(0) ≥ *μ*_1_(0) ≥ *μ*_1_(*x*) = *μ*(*x*) implies *μ*(0) ≥ *μ*(*x*) for any *x* ∈ *X*. Similarly, if *μ*_2_(*x*) ≥ *μ*_1_(*x*), then *μ*(0) ≥ *μ*(*x*) for any *x* ∈ *X*. Hence *μ* is a fub over (*X*, ∗, 0)

Assume that *μ*(*x*∗*y*) = *μ*(*y*∗*x*) = *μ*(0). Then *μ*_1_(*x*∗*y*)∨*μ*_2_(*x*∗*y*) = *μ*(*x*∗*y*) = *μ*(0) =  *μ*_1_(0)∨*μ*_2_(0) = *μ*_2_(0). If *μ*_2_(*x*∗*y*) < *μ*_2_(0), then *μ*_1_(*x*∗*y*) = *μ*_2_(0) and *μ*_1_(0) ≥ *μ*_1_(*x*∗*y*) = *μ*_2_(0), which is a contradiction. Hence *μ*_2_(*x*∗*y*) ≥ *μ*_2_(0); that is, *μ*_2_(*x*∗*y*) = *μ*_2_(0). Similarly, *μ*(*y*∗*x*) = *μ*(0) implies *μ*_2_(0) = *μ*_2_(*y*∗*x*). Hence *μ*_2_(*x*∗*y*) = *μ*_2_(*y*∗*x*) = *μ*_2_(0). Since *μ*_2_ has the condition (*A*)*, we have *μ*_2_(*x*) = *μ*_2_(*y*) = *μ*_2_(0) and so *μ*(*x*) = *μ*(0). Similarly, we obtain *μ*(*y*) = *μ*(0). Thus *μ* has the condition (*A*)*.

**Example**

(a) Suppose *A* and *B* are subsets of *X* such that *A*∖*B*, *A*∩*B*, and *B*∖*A* are nonempty. Let 0 ∈ *A*∩*B* and let *μ*_1_ : = *χ*_*A*_, *μ*_2_ : = *χ*_*B*_, where *x* ∈ *A*∖*B*, *y* ∈ *B*∖*A*, and (*X*, ∗) is the left-zero-semigroup. It follows that *μ*(*x*∗*y*) = *μ*_1_(*x*∗*y*)∨*μ*_2_(*x*∗*y*) = *μ*(*x*) = *μ*_1_(*x*)∨*μ*_2_(*x*) = 1∨0 = 1 and that *μ*(*y*∗*x*) = 1 = *μ*(*y*), *μ*(0) = 1 = *μ*_1_(1) = *μ*_2_(1). Hence *μ*(*x*) = *μ*(*y*) = *μ*(0); that is, the condition (*A*)* holds.

(b) Let (*R*, −, 0) be the set of all real numbers equipped with subtraction and the zero element 0. Let *μ*_1_(*x*): = 1 + *x* − ⌈*x*⌉ and *μ*_2_(*x*): = 1 + ⌊*x*⌋ − *x*. If *x* ∈ *Z*, then *x* = ⌈*x*⌉ = ⌊*x*⌋ and hence *μ*_1_(*x*) = *μ*_2_(*x*) = 1 = *μ*(0). If *x* = *n* + *α* where *n* ∈ *Z*, 0 < *α* < 1/2, then *μ*_1_(*x*) = *α* and *μ*_2_(*x*) = 1 − *α*. Hence *μ*(*x*) = *μ*_1_(*x*)∨*μ*_2_(*x*) = 1 − *α*. If *x* = *n* + *β* where *n* ∈ *Z*, 1/2 < *β* < 1, then *μ*_1_(*x*) = *β* and *μ*_2_(*x*) = 1 − *β*. Hence *μ*(*x*) = *μ*_1_(*x*)∨*μ*_2_(*x*) = *β*. Assume *μ*(*x* − *y*) = *μ*(*y* − *x*) = *μ*(0). Since *μ*(0) = 1, we have *x* − *y*, *y* − *x* ∈ *Z*, say *y* − *x* = *n* ∈ *Z*. If we let *x* : = *m* + *γ* where *m* ∈ *Z*, 0 < *γ* < 1, then *y* = *x* + *n* = (*n* + *m*) + *γ*. It follows that *μ*(*x*) = *μ*(*y*) has the value either *γ* or 1 − *γ*. Hence *μ* satisfies the condition (*A*), but not the condition (*A*)*.

Note that, in Example 9 (b), we know that *μ*_1_(0) = *μ*_2_(0) = 1. This shows that the condition *μ*_1_(0) < *μ*_2_(0) is a very necessary condition in Theorem 8.

## 4. Either-or in Groupoids

**Definition**

Let (*X*, ∗) be a groupoid and let *∅* ≠ *U*⊆*X*. Then *U* is said to be* either-or* if *x*∗*y* ∈ *U*, *y*∗*x* ∈ *U* implies either {*x*, *y*}⊆*U* or {*x*, *y*}⊆*U* ^*c*^ for any *x*, *y* ∈ *X*.

**Proposition**

Suppose that *μ* is a *fub* over a groupoid (*X*, ∗, 0) and that *μ* satisfies the condition (*A*). Let Ker⁡*μ* : = {*x* ∈ *X* | *μ*(*x*) = *μ*(0)}. Then Ker⁡*μ* is an either-or subset of *X*.

**Proof**

Let *x*∗*y*, *y*∗*x* ∈ Ker⁡*μ* for any *x*, *y* ∈ *X*. Then *μ*(*x*∗*y*) = *μ*(0) = *μ*(*y*∗*x*). Since *μ* satisfies the condition (*A*), we have *μ*(*x*) = *μ*(*y*). Thus, also, if *x* ∈ Ker⁡*μ*, then *y* ∈ Ker⁡*μ*, and if *x* ∉ Ker⁡*μ*, then *y* ∉ Ker⁡*μ*. Therefore Ker⁡*μ* is an either-or subset of *X*.

Note that 0 ∈ Ker⁡*μ* in Proposition 11.

**Definition**

An either-or subset *U* is said to be* with alternative* if there are elements {*x*, *y*}⊆*U*, {*u*, *v*}⊆*U* ^*c*^ such that {*x*∗*y*, *y*∗*x*}⊆*U*, {*u*∗*v*, *v*∗*u*}⊆*U*.

**Example**

If (*Z*, +) is the groupoid of all integers with respect to the usual addition and if *U* : = 2*Z* is the set of all even integers, then *U* is an either-or subset of *X*. In fact, let *x* + *y* = *y* + *x* = 2*u* ∈ *U* and *x* = 2*v*, where *u*, *v* ∈ *Z*. Then *y* = 2*u* − 2*v* = 2(*u* − *v*) ∈ *U*. Thus, *U* is an either-or subset of (*Z*, +). On the other hand, *U* ^*c*^ consists of all odd integers. Now if *x* + *y* = *y* + *x* is odd for any *x*, *y* ∈ *Z* and if *x* is odd, then *y* = (*x* + *y*) − *x* is even. If *x* is even, then *y* is odd. Hence *U* ^*c*^ fails to be an either-or subset of (*Z*, +). The subset *U* = 2*Z* of (*Z*, +) is an either-or subset with alternative. Both *Z* and *Z* ^*c*^ = *∅* are either-or subsets, but without alternative.

**Proposition**

Let 0 ∈ *U* be a nonempty subset of a groupoid (*X*, ∗). Suppose that *U* is an either-or subset of the groupoid (*X*, ∗) and that *μ* = *χ*_*U*_ is the characteristic function of *U*; that is, *μ*(*x*) = 1 if *x* ∈ *U* and *μ*(*x*) = 0 otherwise. Then *μ* satisfies the condition (*A*).

**Proof**

Let 0 ∈ *U* be a “selected” element; that is, *μ*(0) = 1. Then *μ*(*x*∗*y*) = *μ*(*y*∗*x*) = *μ*(0) implies *x*∗*y*, *y*∗*x* ∈ *U*. Since *U* is an either-or subset of the groupoid (*X*, ∗), we have either {*x*, *y*}⊆*U* or {*x*, *y*}⊆*U* ^*c*^; that is, *μ*(*x*) = *μ*(*y*) = 1 or *μ*(*x*) = *μ*(*y*) = 0. In any case *μ*(*x*) = *μ*(*y*) and the condition (*A*) holds.

Let (*X*, ∗) be a groupoid and let *∅* ≠ *S*⊆*X*. Let EO(*S*) be the collection of all either-or subsets of the groupoid (*X*, ∗), sometimes denoted as EO(*S*; (*X*, ∗)), which contain the subset *S* of *X*. Then, since (*X*, ∗) is an either-or subset of (*X*, ∗), it follows that EOR(*S*): = ∩_*i*_{*U*_*i*_ | *U*_*i*_ ∈ EO(*S*; (*X*, ∗))}⊇*S*.

**Proposition**

Let (*X*, ∗) be a groupoid and let *∅* ≠ *S*⊆*U*. Then *EOR*(*S*; (*X*, ∗)) is an either-or subset of (*X*, ∗) containing *S*.

**Proof**

Let *x*, *y* ∈ *X* such that *x*∗*y*, *y*∗*x* ∈ EOR(*S*; (*X*, ∗)). If {*x*, *y*}⊆*U* ^*c*^ for some either-or subset *U* of (*X*, ∗) which contains *S*, then EOR(*S*; (*X*, ∗))⊆*U* implies *U* ^*c*^⊆EOR(*S*, (*X*, ∗))^*c*^ and {*x*, *y*}⊆EOR(*S*, (*X*, ∗))^*c*^. If this is not the case, then {*x*, *y*} ⊈ *U* ^*c*^ for any either-or subset *U* of (*X*, ∗) containing *S*, whence {*x*, *y*}⊆*U*. Thus {*x*, *y*}⊆EOR(*S*; (*X*, ∗)). This shows that EOR(*S*; (*X*, ∗)) is an either-or subset of (*X*, ∗).

We have the following corollaries.

**Corollary**

Let (*X*, ∗) be a groupoid. If *∅* ≠ *S*⊆*T*, then *EOR*(*S*; (*X*, ∗))⊆*EOR*(*T*; (*X*, ∗)).

**Corollary**

Let (*X*, ∗) be a groupoid. Then we have

(7) ∅=EOR(∅;(X,∗))⊆EOR({0};(X,∗))⊆⋯⊆EOR(X;(X,∗))=X.

**Example 18**

(a) Let (*X*, ∗) be the left-zero-semigroup; that is, *x*∗*y* = *x* for all *x*, *y* ∈ *X*, and let 0 be a fixed element for which *μ*(0) ≥ *μ*(*x*) for all *x* ∈ *X*, where *μ* : *X* → [0,1] is a fuzzy subset of *X*. Then *μ*(*x*∗*y*) = *μ*(*x*) = *μ*(*y*∗*x*) = *μ*(*y*) = *μ*(0) implies *μ*(*x*) = *μ*(*y*) = *μ*(0) and *μ* satisfies the condition (*A*)*. If *U* is any nonempty subset of *X*, then *x*∗*y*, *y*∗*x* ∈ *U* implies {*x*, *y*}⊆*U*, so that *U* is an either-or subset without alternative. Hence EOR(*U*; (*X*, ∗)) = *U* for any *∅* ≠ *U*⊆*X* whatever. Of course, if (*X*, ∗, 0) is the right-zero-semigroup, that is, *x*∗*y* = *y* for any *x*, *y* ∈ *X*, then the same conclusions hold.

(b) Let (*Z*, +, 0) be the group of all integers with respect to addition. We claim that *nZ* = {*nx* | *x* ∈ *Z*} is an either-or subset of (*Z*, +, 0). Given *n* ∈ *Z*, if *n* = 0, and if *x* + *y*, *y* + *x* ∈ {0} and *x* = 0, then *y* = 0 and hence {*x*, *y*} = {0}⊆{0}. On the other hand, if *x* ≠ 0, then *y* = −*x* implies {*x*, *y*} = {*x*, −*x*}⊆{0}^*c*^ = *Z* − {0}. Hence {0} = 0*Z* is an either-or subset of the groupoid (*Z*, +, 0). Let *n* ≠ 0. If *x* + *y* = *y* + *x* = *nu*, and if *x* = *nv*, then *y* = (*x* + *y*) − *x* = *nu* − *nv* = *n*(*u* − *v*); that is, *y* ∈ *nZ* as well. Thus {*x*, *y*}⊆*nZ*. Assume that *x* ∉ *nZ*; that is, *x* = *nu*′ + *α* for some *u*′ ∈ *Z* and *α* ∈ *Z* such that 0 < *α* < *n*. Then *y* = *nu* − *x* = *n*(*u* − *u*′) − *α* ∉ *nZ*. Hence {*x*, *y*}⊆(*nZ*)^*c*^.

We claim that EOR({*n*}; (*Z*, +)) = *nZ* for all *n* ∈ *Z*. Since {0} is the smallest either-or subset of (*Z*, +), EOR({0}; (*Z*, +)) = {0} = 0*Z*. Let *n* ≠ 0 in *Z*. Then 0 ∈ EOR({*n*}; (*Z*, +)). In fact, if *U*_*i*_ is an either-or subset of (*Z*, +) containing *n*, then either {*n*, 0}⊆*U*_*i*_ or {*n*, 0}⊆*U*_*i*_^*c*^, since *n* + 0 = 0 + *n* = *n* ∈ *U*_*i*_. It follows that 0 ∈ *U*_*i*_ for all either-or subset *U*_*i*_ of (*Z*, +); that is, 0 ∈ EOR({*n*}; (*Z*, +)).

We claim that −*n* ∈ EOR({*n*}; (*Z*, +)). If *U*_*i*_ is an either-or subset of (*Z*, +), either {*n*, −*n*}⊆*U*_*i*_ or {*n*, −*n*}⊆*U*_*i*_^*c*^ for any either-or subset *U*_*i*_ of (*Z*, +), since *n* + (−*n*) = (−*n*) + *n* = 0 ∈ *U*_*i*_. Since *n* ∈ *U*_*i*_, we have −*n* ∈ *U*_*i*_ for all either-or subset *U*_*i*_. This proves that −*n* ∈ EOR({*n*}; (*Z*, +)). Hence {−*n*, 0, *n*}⊆EOR({*n*}; (*Z*, +)).

Assume that *B* is an either-or subset of (*Z*, +) containing *n*. Then it is easy to see that 0, −*n* ∈ *B*. If we let *x* : = *n*, *y* : = −2*n*, then *x* + *y* = *y* + *x* = −*n* ∈ *B*. Since *x* = *n* ∈ *B* and *B* is an either-or subset of (*Z*, +), we obtain −2*n* = *y* ∈ *B*. Similarly, if we let *x* = −*n* and *y* : = 2*n*, then we obtain 2*n* = *y* ∈ *B*. Similarly, we obtain ±3*n*, ±4*n*,…, ∈*B*. Thus *nZ*⊆*B*; that is, *B* is an either-or subset of (*Z*, +) containing *nZ*. It follows that *nZ* = ∩{*U*_*i*_ | *U*_*i*_ :  either-or  subset  containing  *n*} = EOR({*n*}; (*Z* , +)).

(c) Let *N* : = {1,2, 3,…} and let *a* ∈ *N*. If *x* + *y* = *a*, then *x* < *a*, *y* < *a* and thus {*a*} is an either-or subset of *N* without alternative, since {*x*, *y*}⊆{*a*}^*c*^. If *U*_*a*_^*k*^ : = {*a*, 2*a*,…, *ka*} for any *a* ∈ *N*, let *x* + *y* = *y* + *x* ∈ *U*_*a*_^*k*^. Then there exists *h* ∈ *N* such that *x* + *y* = *ha*, *h* ≤ *k*. If *x* = *ja*, *j* < *h*, then *y* = *ha* − *x* = (*h* − *j*)*a* ∈ *U*_*a*_^*k*^. Hence {*x*, *y*}⊆*U*_*a*_^*k*^. If *x* ∉ *U*_*a*_^*k*^, then *y* ∉ *U*_*a*_^*k*^ and {*x*, *y*}⊆(*U*_*a*_^*k*^)^*c*^, so that *U*_*a*_^*k*^ is an either-or subset of *N* with alternative if *k* ≥ 2.

**Proposition**

Let (*X*, ∗) be a groupoid and let *U*⊆*X* such that *x*∗*y*, *y*∗*x* ∈ *U* implies *x* = *y*. Then *U* is an either-or subset of (*X*, ∗).

**Proof**

Let *x*∗*y*, *y*∗*x* ∈ *U* for any *x*, *y* ∈ *X*. By assumption, we have *x* = *y* and so {*x*, *y*} = {*x*}. Hence either {*x*}⊆*U* or {*x*}⊆*U* ^*c*^. Thus *U* is an either-or subset of *X*.

We call such a subset *U* of *X* a* strong either-or subset* of (*X*, ∗).

**Example**

(a) Let (*X*, ∗, *f*) be a leftoid, and let *U* = {*a*} be a singleton. If *x*∗*y* = *f*(*x*) ∈ *U* and *y*∗*x* = *f*(*y*) ∈ *U*, then *f*(*y*) = *f*(*x*) = *a*. Hence, if |*f* ^−1^(*a*)| = 1, then *x* = *y*, and *U* is a strong either-or subset of (*X*, ∗).

(b) Let *N* be the set of all natural numbers and “+” be the usual addition on *N*. If *U* : = {2}, then *U* is a strong either-or subset of *N*. In fact, if *x* + *y* = *y* + *x* ∈ *U*, then *x* + *y* = *y* + *x* = 2 and hence *x* = *y* = 1.

**Proposition**

Let (*X*, ∗, 0) be a groupoid and let *U* be a strong either-or subset of *X* with 0 ∈ *U*. If *μ* : = *χ*_*U*_ is the characteristic function of *U*, then *μ* is a *fub* over (*X*, ∗, 0). Furthermore, *μ* satisfies the condition (*B*) and Ker⁡*μ* = *U*.

**Proof**

Since 0 ∈ *U*, we have *μ*(0) = 1 ≥ *μ*(*x*) for all *x* ∈ *X* and *μ* is a fub over (*X*, ∗, 0). Let *μ*(*x*∗*y*) = *μ*(*y*∗*x*) = *μ*(0) for any *x*, *y* ∈ *X*. Since *μ*(0) = 1, we have *x*∗*y*, *y*∗*x* ∈ *U*. Hence *x* = *y*, since *U* is a strong either-or subset of *X*. Therefore *μ* satisfies the condition (*B*) and in that case Ker⁡*μ* = *μ* ^−1^(*μ*(0)) = *U*.

**Proposition**

Let (*X*, ∗, *e*) be a group all of whose elements have finite order and let *μ* : *X* → [0,1] be a fuzzy subgroup. Then *μ* has the condition (*A*) and Ker⁡*μ* is an either-or subset of *X*.

**Proof**

Let *μ* be a fuzzy subgroup of *X*. Then *μ*(*x*∗*y*) ≥ min⁡{*μ*(*x*), *μ*(*y*)} for any *x*, *y* ∈ *X*. Thus *μ*(*x* ^2^) = *μ*(*x*∗*x*) ≥ *μ*(*x*) and *μ*(*x* ^*n*^) ≥ min⁡{*μ*(*x* ^*n*−1^), *μ*(*x*)}. By induction *μ*(*x* ^*n*−1^) ≥ *μ*(*x*) implies *μ*(*x* ^*n*^) ≥ *μ*(*x*), so that *μ*(*e*) = *μ*(*x* ^*n*^) ≥ *μ*(*x*) implies *μ*(*e*) ≥ *μ*(*x*) for all *x* ∈ *X*; that is, *μ* is a fub over (*X*, ∗, *e*).

If Ker⁡*μ* = *μ* ^−1^(*μ*(*e*)) = *U*, then *μ*(*x*∗*y*) = *μ*(*y*∗*x*) = *μ*(*e*) implies *μ*((*x*∗*y*)∗*y* ^−1^) = *μ*(*x*) ≥ min⁡{*μ*(*x*∗*y*), *μ*(*y* ^−1^)) and hence *μ*(*x*) ≥ *μ*(*y* ^−1^) ≥ *μ*(*y*), so that *μ*(*x*) = *μ*(*y*); the condition (*A*) holds for *μ*, and Ker⁡*μ* is an either-or subset of (*X*, ∗, *e*).

## 5. Fuzzy Upper Bounds in Bin(*X*)

**Theorem**

Let *μ* be a *fub* over (*X*, ∗, 0) with the condition (*A*) and a *fub* over (*X*, •, 0) with the condition (*A*)*. If (*X*, □): = (*X*, ∗)□(*X*, •), then *μ* is a *fub* over (*X*, □, 0) with the condition (*A*)*.

**Proof**

Assume that *μ*(*x*□*y*) = *μ*(*y*□*x*) = *μ*(0) for any *x*, *y* ∈ *X*. Then *μ*((*x*∗*y*)•(*y*∗*x*)) = *μ*((*y*∗*x*)•(*x*∗*y*)) = *μ*(0) implies *μ*(*x*∗*y*) = *μ*(*y*∗*x*) = *μ*(0), so that *μ*(*x*) = *μ*(*y*) = *μ*(0). Hence *μ* satisfies the condition (*A*)* over (*X*, □, 0).

**Corollary**

Let *μ* be a *fub* over both (*X*, ∗, 0) and (*X*, •, 0) with the condition (*A*)*. If (*X*, □): = (*X*, ∗)□(*X*, •), then *μ* is an *lub* over (*X*, □, 0) with the condition (*A*)*.

**Proof**

Straightforward.

Let *X* be a nonempty set. If 〈*μ*; (*A*)〉 and 〈*μ*; (*A*)*〉 are the collections of groupoids (*X*, ∗, 0) such that *μ* satisfies the condition (*A*) (resp., (*A*)*), then 〈*μ*; (*A*)*〉⊆〈*μ*; (*A*)〉 and

(8) 〈μ;(A)〉□〈μ;(A)∗〉⊆〈μ;(A)〉,〈μ;(A)∗〉□〈μ;(A)∗〉⊆〈μ;(A)∗〉;

that is, (〈*μ*; (*A*)*〉, □) is a subsemigroup of (Bin(*X*), □).

**Theorem**

Let *μ* be a *fub* over (*X*, ∗, 0) with the condition (*B*), and let *μ* be a fub over (*X*, •, 0) with the condition (*A*)*. If (*X*, □): = (*X*, ∗)□(*X*, •), then *μ* is a *fub* over (*X*, □, 0) with the condition (*B*).

**Proof**

Assume that *μ*(*x*□*y*) = *μ*(*y*□*x*) = *μ*(0) for any *x*, *y* ∈ *X*. Then *μ*((*x*∗*y*)•(*y*∗*x*)) = *μ*((*y*∗*x*)•(*x*∗*y*)) = *μ*(0). Since *μ* satisfies the condition (*A*)* over (*X*, •, 0), we have *μ*(*x*∗*y*) = *μ*(*y*∗*x*) = *μ*(0). Since *μ* has the condition (*B*) over (*X*, ∗, 0), we obtain *x* = *y*, proving the theorem.

## 6. Fuzzy-*d*-Subsets in Groupoids 

Given a groupoid (*X*, ∗, 0), the following are interesting properties in fuzzy subgroupoids *μ* : *X* → [0,1]:

1. *μ*(*x*∗*x*) = *μ*(0) for all *x* ∈ *X*,
2. *μ*(0∗*x*) = *μ*(0) for all *x* ∈ *X*,
3. if *μ*(*x*∗*y*) = *μ*(*y*∗*x*) = *μ*(0), then *μ*(*x*) = *μ*(*y*) for all *x*, *y* ∈ *X*.

**Definition**

A fub  *μ* over a groupoid (*X*, ∗) is called a* fuzzy-d-subset* of the groupoid (*X*, ∗, 0) if it satisfies conditions (1), (2), and (3).

**Example**

(a) Let *X* : = *R* be the set of all real numbers and let “+” be the usual addition on *R*. Then every fuzzy-*d*-subset *μ* of (*X*, +, 0) is constant. In fact, for all *x* ∈ *X*, we have *μ*(0) = *μ*(*x* + *x*) = *μ*(2*x*). If we let *y* : = 2*x*, then *μ*(*y*) = *μ*(0) for all *y* ∈ *X*; that is, *μ* is a constant function on *X*.

(b) Let (*X*, ∗, 0) be a group with identity 0. Let *μ* be a fuzzy subset of *X* with (2); then *μ*(*x*) = *μ*(0∗*x*) = *μ*(0) for all *x* ∈ *X*, whence *μ* is a constant function. Therefore *μ* is a fuzzy-*d*-subset of (*X*, ∗, 0).

**Proposition**

Let (*X*, ∗, 0) be a *d*-algebra. If *μ* = *χ*_{0}_ is the characteristic function of {0}; that is, *μ*(0) = 1 and *μ*(*x*) = 0 otherwise, then *μ* is a fuzzy-*d*-subset of *X* and Ker⁡*μ* = {0}.

**Proof**

Let *x*, *y* ∈ *X*. Then *μ*(*x*∗*x*) = *μ*(0), *μ*(0∗*x*) = *μ*(0). If *μ*(*x*∗*y*) = *μ*(*y*∗*x*) = *μ*(0), then *x*∗*y* = *y*∗*x* = 0. Since (*X*, ∗, 0) is a *d*-algebra, we obtain *x* = *y*. Hence *μ*(*x*) = *μ*(*y*). This shows that *μ* is a fuzzy-*d*-subset of (*X*, ∗, 0) and Ker⁡*μ* = *μ* ^−1^(*μ*(0)) = *μ* ^−1^(1) = {0}.

**Proposition**

Let *S* be an either-or subset of a groupoid (*X*, ∗, 0) such that Δ(*X*, ∗) : = {*x*∗*x* | *x* ∈ *X*} and Δ_0_(*X*, ∗): = {0∗*x* | *x* ∈ *X*} are subsets of *S*. Let *μ* : = *χ*_*S*_ be the characteristic function of *S* and 0 ∈ *S*. Then *μ* is a fuzzy-*d*-subset of (*X*, ∗, 0).

**Proof**

Since 0 ∈ *S*, we have *μ*(0) = 1 ≥ *μ*(*x*) for all *x* ∈ *X*. Since Δ(*X*, ∗), Δ_0_(*X*, ∗)⊆*S*, we obtain *μ*(*x*∗*x*) = *μ*(0), *μ*(0∗*x*) = *μ*(0). Assume that *μ*(*x*∗*y*) = *μ*(*y*∗*x*) = *μ*(0). Then *x*∗*y*, *y*∗*x* ∈ *S*. Since *S* is an either-or set over (*X*, ∗, 0), either {*x*, *y*}⊆*S* or {*x*, *y*}⊆*S* ^*c*^. It follows that either *μ*(*x*) = *μ*(*y*) = 1 or *μ*(*x*) = *μ*(*y*) = 0. Hence *μ* is a fuzzy-*d*-subset of (*X*, ∗, 0).
